# Exploring the Use of Pre-exposure Prophylaxis (PrEP) for HIV Prevention Among High-Risk People Who Use Drugs in Treatment

**DOI:** 10.3389/fpubh.2018.00195

**Published:** 2018-07-13

**Authors:** Roman Shrestha, Michael Copenhaver

**Affiliations:** ^1^Department of Allied Health Sciences, University of Connecticut, Storrs, CT, United States; ^2^Institute for Collaboration on Health, Intervention, & Policy, University of Connecticut, Storrs, CT, United States

**Keywords:** HIV prevention, pre-exposure prophylaxis, people who use drugs, substance abuse, methadone maintenance program

## Abstract

**Introduction:** Despite unequivocal evidence supporting the use of pre-exposure prophylaxis (PrEP), its scale-up has been gradual overall, and nearly absent among people who use drugs (PWUD). In the present study, we implemented the use of PrEP, as a part of an integrated HIV prevention approach, and explored the experiences and attitudes related to PrEP use among PWUD.

**Methods:** Between September 2016 and July 2017, we recruited 40 HIV-uninfected, methadone-maintained people, who reported HIV-risk behaviors, and were currently taking PrEP. We conducted both quantitative and in-depth semi-structured qualitative interviews that primarily focused on experiences, attitudes, acceptability, disclosure status, risk compensation-related attitudes, and barriers related to PrEP adherence.

**Results:** Results showed that participants were highly satisfied and perceived PrEP as valuable and acceptable for HIV prevention. Participants reported high adherence to PrEP. The most highly endorsed facilitators to PrEP adherence were use of memory aids, no out-of-pocket cost, perceived benefit, and support from social network. The barriers to adherence included side-effects, stigmatization, requirement of daily dosing, and accessibility of PrEP services. Additionally, participants expressed disagreement with the overall risk compensation-related attitudes (i.e., decreased personal concern about engaging in HIV risk behavior due to their perception that PrEP is now fully protecting them from contracting HIV) and indicated no increased engagement in risk behaviors while on PrEP.

**Conclusions:** The results from the current study provide preliminary evidence supporting the successful integration of PrEP within the substance abuse treatment setting, where high risk PWUD are concentrated.

## Introduction

With the rise of illicit drug use in the United States (US), people who use drugs (PWUD) continue to remain a population at high-risk for HIV infection (1–3). The availability of pre-exposure prophylaxis (PrEP) is emerging as an important tool for curtailing the HIV epidemic and a recommended element of integrated HIV prevention approaches (4–6). Recent large-scale PrEP trials have shown PrEP to be safe, well tolerated, and highly efficacious in reducing the risk of HIV acquisition in individuals at increased risk for infection (7–11). Based on these trials, the Centers for Disease Control and Prevention (CDC) made recommendations and provided clinical practice guidelines on the use of PrEP for HIV prevention among most-at-risk populations, including PWUD (12).

In the US, the CDC estimates that PrEP could benefit 1 in 4 adult men who have sex with men (MSM), 1 in 5 people who inject drugs (PWID: who are subset of PWUD group), and 624,000 heterosexually active adults, three-quarters of whom are women (13). As such, PrEP implementation is underway around the world, particularly among MSM. However, the scale-up of PrEP among even very high-risk PWUD is nearly absent, despite being ideal candidates and expressing strong willingness to use PrEP (14–17). As indicated in the original PrEP trial among PWID (11), common drug treatment setting (e.g., methadone maintenance program: MMP) play a key role in the adoption and delivery of PrEP among PWUD (18). Particularly in the context of MMP, the requirement for routine counseling and coordination of care readily support the integration of PrEP and facilitate improvement of the PrEP continuum of care (cascade) among this underserved group (19). Furthermore, the requirement for daily contact with service providers for methadone administration may allow the counselors/nurses to monitor clients' adherence to medication through directly observed therapy (DOT).

Considering these factors, we implemented the use of PrEP as a part of an integrated primary HIV prevention approach among PWUD who exhibited high HIV transmission risk behaviors (both sex- and drug-related) in the context of an MMP (18, 20). In the present study, we explored the experiences, attitudes, acceptability, disclosure status, risk compensation-related attitudes, and barriers to adherence among PWUD using PrEP. Such information is crucial to optimize PrEP programs in addiction treatment, where high risk PWUD are concentrated, and are ideal settings to implement primary HIV prevention interventions among PWUD.

## Methods

### Study setting and participants

We used purposive sampling to recruit participants who met the eligibility criteria. A total of 40 participants were recruited from an inner-city MMP in New Haven, Connecticut if they (i) were age ≥18 years, (ii) were confirmed HIV-negative and on PrEP, (iii) reported drug- (e.g., sharing of injection equipment) or sex-related (e.g., condomless sex, multiple sex partners) HIV risk behaviors in the past 6 months, and (iv) enrolled in MMP.

### Procedures

Between September 2016 and July 2017, we conducted both quantitative and in-depth, semi-structured qualitative interviews among PWUD who were using PrEP. Participants were recruited via flyers, peers, word-of-mouth, and direct referral from counselors at the MMP. Interested individuals were screened for HIV transmission risk behavior by the research assistant. Those who reported high-risk behavior and interest in taking PrEP in the screening form, but not already on PrEP (38 out of 40 participants), were referred for additional screening (i.e., PrEP eligibility) (12) by an onsite primary care physician for PrEP prescription. Those who chose to initiate PrEP were then enrolled in the study and provided written consent prior to completing the assessments. Participants were reimbursed for their time ($45). The study protocol was approved by the Institutional Review Board at the University of Connecticut and received board approval from the methadone clinic.

### Assessments

#### Quantitative assessment

All quantitative measures were assessed using an audio computer-assisted self-interview (ACASI). In addition to demographic and social characteristics, we assessed homeless status, current methadone dose, perceived HIV risk, length of PrEP use, PrEP prescription venue, and PrEP disclosure status. Other measures are described as follows:

##### HIV risk behaviors

The HIV risk assessment, adapted from National Institute on Drug Abuse (NIDA)'s Risk Behavior Assessment (21) was used to measure several aspects of HIV risk behaviors, including a measurement of “any” high risk behavior (sexual or drug-related) as well as measurements of event-level (i.e., partner-by-partner) behaviors. For example, “*Have you ever used illicit drugs?”*; “*Have you injected illicit drugs in the last 30 days?”* Responses were reported using a “yes” or “no”.

##### PrEP adherence

PrEP adherence was assessed using a self-reported, validated 3-item scale developed by Wilson et al. (22) Items included: “*In the last 30 days, on how many days did you miss at least one dose of your PrEP medication?”; “In the last 30 days, how often did you take your PrEP medication in the way you were supposed to?”; and “In the last 30 days, how good a job did you do at taking your PrEP medication in the way you were supposed to?*” Summary scales were calculated as the mean of the three individual items with higher score indicating better adherence (0–100) (22).

##### Acceptability of PrEP use

Acceptability of PrEP use was assessed using a series of 8-item acceptability rating profile (23). Participants used a five-point Likert scale (0 = *strongly disagree* to 4 = *strongly agree*) to rate the extent to which they agreed with each acceptability statement. For example, “*PrEP is useful for individuals who engage in high risk behaviors (e.g., unsafe sex, needle sharing),” “I would be willing to recommend PrEP to my friends who may benefit from it.”* An intervention acceptability score was calculated by first adding the scores and then converting into a score on the 0–100 scale with higher values indicating greater acceptability (α = 0.797).

##### Risk compensation related attitudes

Risk Compensation related attitudes was assessed using the Treatment-Related Reduced HIV Risk measure (24), an 8-item scale adapted for PrEP use that assesses decreased personal concern about engaging in unsafe sex and the potential for infecting others because of PrEP use. For example, “*Because I am taking PrEP, I am less concerned about becoming HIV positive,” “I am more willing to take a chance of getting infected now that I am taking PrEP.”* Responses were reported using a “yes” or “no.” The scale was internally reliable in this sample (α = 0.752).

#### Qualitative assessment

All qualitative interviews were semi-structured around a set of carefully predetermined interview guides (Table 1). These guides were brief, semi-structured to encourage free-flowing discussion between interviewer and participant. Interviews explored overall experiences taking PrEP to prevent HIV, facilitators and barriers to PrEP use, and engagement in risk behaviors while on PrEP. All qualitative interviews were audiotaped with participants' permission and transcribed verbatim.

**Table 1:** Interview guides.

(1) How do you feel about using PrEP as a prevention method?
(2) How do you feel about disclosing that you are currently taking PrEP to others, such as your partner, family, relatives, etc.?
(3) What are some of the challenges or concerns of taking PrEP as prescribed?
(4) What are some of the facilitators of taking PrEP as prescribed?
(5) What do you feel about using condom or injecting since you're on PrEP now?
(6) Would you recommend PrEP to your friends you may benefit from its use?
(7) What do you think would be helpful for us to consider in terms of better designing the PrEP program?


### Data analyses

Analyses of quantitative data were performed in SPSS. Descriptive statistics were performed to provide a demographic, sexual and substance use profile of study participants. Means and standard deviations (continuous variables) and frequencies and percentages (categorical variables) were derived to assess participants' adherence, acceptability and risk compensation regarding the use of PrEP for HIV prevention. Analysis of qualitative data followed a thematic analysis approach, applying several qualitative data analysis procedures (e.g., inductive analysis, cross-case analysis). In the initial inductive analysis, emergent themes and patterns were identified directly in the transcripts. The coding process was completed for each transcript and a master list of themes was compiled to reflect overarching themes. Two research team members met regularly to become acquainted with participant narratives, to contextualize differences, to build consensus, and to cross-case analysis decisions of emerging themes.

## Results

### Participant characteristics

Participants were mostly in their mid-40s (mean = 44.8), male (55.0%), White (57.5%), and single (47.5%). Most participants identified as heterosexual (77.5%) and graduated from high school (75.0%). Only 7.5% of the participants reported being currently employed and 77.5% were earning less than $10,000 per year. Almost one-third of the participants (27.5%) reported to have been homeless in the past 30 days. All participants were enrolled in an inner-city MMP and were maintained on a stable dose of methadone (mean = 78.5 mg) (Table 2).

**Table 2:** Characteristics of the participants.

**Variable**	**Frequency**	**%**
Age: Mean (±*SD*)	44.8 (±11.8)	
**GENDER**		
Male	22	55.0
Female	18	45.0
**SEXUAL ORIENTATION**		
Heterosexual	31	77.5
Homosexual, gay, or lesbian	3	7.5
Bisexual	6	15.0
**ETHNICITY**		
White	23	57.5
African American	13	32.5
Hispanic or Latino	3	7.5
Other	1	2.5
**MARITAL STATUS**		
Married	12	30.0
High school graduate	30	75.0
Employed	3	7.5
**INCOME**		
< $10,000 USD	31	77.5
$10,000 - $19,999 USD	7	17.5
≥ $20,000 USD	2	5.0
Homeless *(past 30 days)*	11	27.5
Methadone dose, mean (mg)	78.5 (±25.8)	
Injected illicit drugs *(past 30 days)*	29	72.5
Shared injection equipment *(past 30 days)*	25	62.5
Been sexually active *(past 30 days)*	32	80.0
Number of sexual partners *(past 30 days)*	*n = 32*	
1	13	40.6
2–5	15	46.9
≥ 6	4	12.6
Always used condom with casual partner	*n = 32*	
No casual partner	5	15.6
No	13	40.6
Yes	14	43.8
**CURRENT RISK OF GETTING HIV**		
Low	8	20.0
Medium	19	47.5
High	13	32.5

*SD, standard deviation; HRBS, HIV risk-taking behavior scale; PrEP, pre-exposure prophylaxis*.

### Quantitative results

#### Sexual risk and substance use behavior

Majority of the participants (72.5%) reported current drug injection in the past 30 days. Of those, almost two-thirds reported having shared injection equipment. Of those who were sexually active (80.0%), 59.5% reported having sex with more than one sexual partner and 43.8% reported always using condoms with casual sexual partners. Most participants perceived themselves to be at medium to high risk of contracting HIV (80.0%).

#### PrEP adherence and disclosure status

The mean length of participants on PrEP was 36.1 days. Primary care physicians at the substance abuse treatment clinic were noted as the top source of PrEP prescriptions. Participants reported having taken PrEP in the past 30 days with a mean adherence score of 87.6 (±18.6). A considerable proportion of the participants reported to have disclosed their PrEP status to other people (77.5%), and out of these, 67% disclosed their status to their friends, 45.2% to their spouse, followed by their parents (25.8%) and children (6.5%) (Table 3).

**Table 3:** Variables of interest related to PrEP use.

**Variable**	**Frequency**	**%**
Length of PrEP use (days)	36.1 (±28.4)	
**PrEP PRESCRIPTION VENUE**		
Substance abuse treatment clinic	22	55.0
Community health care van	14	35.0
Others	4	10.0
PrEP adherence	87.6 (±18.6)	
**DISCLOSED PrEP STATUS**		
No	9	22.5
Yes	31	77.5
PrEP status disclosed to	*n = 31*	
Spouse	14	45.2
Parents	8	25.8
Children	2	6.5
Friends	21	67.7
Acceptability of PrEP use	77.7 (±13.9)	
Risk compensation-related attitudes	16.7 (±5.2)	

#### Acceptability of PrEP use

Our results showed that participants were satisfied and perceived the use of PrEP for HIV prevention as valuable and acceptable. Mean acceptability (range: 0–100) for the PrEP use was 77.7 (±13.9), which conveys strong agreement among participants about the acceptability of the PrEP use. Additionally, the majority of participants (90.0%) viewed the use of PrEP as beneficial for individuals who engage in high risk behaviors (e.g., condomless sex, sharing of injection equipment). Similarly, the majority of participants indicated that individuals in substance abuse treatment clinics have high risk behaviors that justifies their use of PrEP (92.5%) and expressed a desire to recommend PrEP to others who may benefit from it (92.5%).

#### Risk compensation-related attitudes

In terms of risk compensation-related attitudes (i.e., decreased personal concern about engaging in HIV risk behavior due to their perception that PrEP is now fully protecting them from contracting HIV), a minority of participants expressed agreement on the overall risk compensation-related attitudes scale (16.7 ± 5.2) (Range: 8–40). For example, none of the participants reported that they were willing to take a chance of getting infected because they were taking PrEP. Similarly, very few of the participants expressed concern about having unprotected sex (10.5%) and sharing of injected equipment (7.9%) while on PrEP. When asked if they have already risked getting infected with HIV through sex (8.16%) and unsafe sharing of injection equipment (86.8%) while on PrEP, most participants responded negatively (Table 4).

**Table 4:** Risk compensation-related attitudes (%).

**Variables**	**Yes**	**No**
Because I am taking PrEP, I am less concerned about becoming HIV positive.	23.7	76.3
I am more willing to take a chance of getting infected now that I am taking PrEP.	00.0	100.0
I am a lot less worried about “slipping up” now that PrEP may be taken prior to unprotected sex.	15.8	84.2
I am a lot less worried about “slipping up” now that PrEP may be taken prior to sharing injection equipment.	13.1	86.9
I am less concerned about having unprotected sex now that I am taking PrEP.	10.5	89.5
I am less concerned about sharing needles or works now that I am taking PrEP.	7.9	92.1
I have already risked getting infected with HIV through unsafe sex while taking PrEP.	18.4	81.6
I have already risked getting infected with HIV through sharing of needles or works while taking PrEP.	13.2	86.8

### Qualitative results

Overall, the results from the qualitative interviews identified the following key themes and subthemes regarding participants' attitudes and experiences surrounding PrEP use (Table 5):

**Table 5:** Themes and subthemes identified from qualitative data.

**Themes**		**Participant quotes**
Adherence to PrEP		*I have it right next to my bed and take it every night before I go to sleep, and it's that easy*.
PrEP adherence barriers	*Side-effects*	*So far, I haven't had any issues but I'm a little worried about the long-term side-effects*.
	*Stigma*	*Because many people out there have no idea what it [PrEP] is for, they would not understand, they will probably think I am infected, thus tell others*.
	*Daily dosing*	*It'd have been so much easier if we could take it [PrEP] whenever we needed them*.
	*Access*	*There were only 7 days of medication. Getting there [to the clinic] once a week was a bit hassle. I missed a day or two because of that. They should've given it for the whole 30 days*.
PrEP adherence facilitators	*Use of memory aids*	*I put an alarm in my phone. That's the only way I remember. I get text message, which has been a big help to help me remember to take it [PrEP]*.
	*Cost*	*The Insurance I currently have covered the doctor visit, blood work they did on me, and for this [PrEP] med*.
	*Perceived benefit*	*If there's a medication out there which is 90% effective in reducing HIV, then why not take it [PrEP]*.
	*Social support*	*Actually, it was my wife who asked me to get on the meds [PrEP]. She has HIV, you know, and she reminds me to take it every single day*.
Risk compensation		*It [use of PrEP] makes me feel I need to be more cautious. Actually, I used a condom last week, that was first time in a while*.

#### Adherence to PrEP

When asked about participants' use of PrEP as a HIV prevention method, most indicated that they had positive experiences with PrEP use and only minor adverse clinical outcomes. The majority of the participants reported consistent adherence patterns to reduce their chances of contracting HIV infection:

“*I've been taking it [PrEP] regularly… I have it right next to my bed and take it every night before I go to sleep, and it's that easy… To be honest, I've only missed a couple of days since I started taking it.”*

Overall, participants expressed favorable attitudes toward the use of PrEP for HIV prevention. A few expressed concerns about the complexities related to its use, whereas, others indicated a few factors to facilitate adherence to this medication.

#### PrEP adherence barriers

##### Side-effects

Participants reported several side-effects when using PrEP, including nausea, headache, stomach-area pain, diarrhea, dizziness, tiredness, problems sleeping, loss of appetite, being short of breath or fast breathing, joint or muscle aches, sore throat, and abnormal dreams. Participants described side-effects that emerged shortly after initiating PrEP, but these diminished over time and did not significantly deter them from taking the pills:

“*So far, I haven't had any issues but I'm a little worried about the long-term side-effects… I used to take it in the mornings. I realized that I was feeling dizzy every time after taking it. Then I started taking it before bedtime and I've had no problem since then.”*

##### Stigma

A few participants noted the potential social risks (e.g., discrimination, exclusion, loss of trust) of taking PrEP, and pointed to instances of both experienced stigma and potential situations where stigmatization could occur. Participants reflected on the social consequences of taking PrEP, which included relationship challenges, rumors, and experienced and perceived stigma, due to PrEP users being perceived as having HIV or being illicit drug users:

“*My roommate asked me if I was taking it for HIV. That threw me off… Because many people out there have no idea what it [PrEP] is for, they would not understand, they will probably think I am infected, and tell others.”*

##### Daily dosing

As participants were asked about the challenges or concerns of taking PrEP as prescribed, some brought up the requirement of daily dosing as one of the barriers. They thought that it was hard for them to adhere to it every day, especially when they were actively using drugs. Few participants expressed a preference for intermittent use as it would be a less-burdensome schedule of pill taking:

“*It'd have been so much easier if we could take it [PrEP] whenever we needed them.”*

##### Access

Some participants pointed out concerns about getting refills and having to make regular follow-up visits as barriers to their adherence to PrEP regimen:

“*Well, refills were a problem. There were only 7 days of medication. Getting there [to the clinic] once a week was a bit of a hassle. I missed a day or two because of that. They should've given it [PrEP] for the whole 30 days.”*

#### PrEP adherence facilitators

##### Use of memory aids

Most participants indicated that they had been using memory aids, such as cell phone, pill organizer, post-it notes to help them remember take PrEP medication:

“*I put an alarm in my phone. That's the only way I remember…I get a text message, which has been a big help for me to remember to take it [PrEP].”*

##### Cost

Participants overwhelmingly reported that the cost for PrEP regimen, including co-payments, related clinical, and laboratory services, were fully covered by their insurance, which facilitated adherence to PrEP medication:

“*The insurance I currently have covered the doctor visit, blood work they did on me, and for this [PrEP] med…I heard it's really expensive. Glad I don't have to pay.”*

##### Perceived benefit

Participants frequently indicated that knowing the high efficacy of PrEP in terms of reducing the risk of HIV infection motivated them to continue taking it:

“*If there's a medication out there which is 90% effective in reducing HIV, then why not take it [PrEP]…When I'm using, I don't think I'll worry about condoms, so it's good that to have PrEP.”*

##### Social support

Some participants reported that good support from their family and friends has been influential in facilitating adherence to PrEP:

“*Actually, it was my wife who asked me to get on the meds [PrEP]. She has HIV, you know, and she reminds me to take it every single day.”*

#### Risk compensation

Participants overwhelmingly reported no increases in engagement in HIV risk behaviors. Surprisingly, the majority of participants reported an increased use of condoms while on PrEP:

“*It [use of PrEP] makes me feel I need to be more cautious. Actually, I used a condom last week, that was first time in a while…It makes me think and I've talked to my boyfriend about using condoms. And, it's been working so far.”*

## Discussion

Several important findings were gleaned from this exploratory study that have promising implications for expanding and improving the PrEP continuum of care in settings such as MMPs (19), where PrEP use among PWUD was originally examined (11). The overwhelmingly positive response regarding PrEP use from study participants was encouraging given the prevalence of high HIV risk behaviors among our sample. Such preferential use of PrEP among our sample of PWUD, 80% of whom perceived themselves to be at moderate to high risk of acquiring HIV, shows these individuals' capacity to recognize and respond appropriately to health risks when given appropriate options. In addition to the biomedical prevention benefits of PrEP, the structured nature of MMP context including the requirement for routine behavioral counseling suggests that MMP settings could readily support the integration of PrEP into existing evidenced-based behavioral risk reduction strategies (18). Our findings thus provide reason for optimism about the potential implementation of PrEP services within the substance abuse treatment settings, where high risk PWUD are easily reached, and thus, has a potential to have profound impact on HIV prevention efforts among PWUD.

Some prescribers and clinicians have raised concerns related to potential low adherence rates leading to low effectiveness of PrEP use and drug resistance among PWUD (18, 25). Participants in the current study, however, reported high adherence to daily PrEP use, as in the original PrEP trial in PWID in MMP (11). As suggested in prior research, adherence might have been related to participants' self-assessed risk of HIV infection (26). It is encouraging to see such patterns among our sample as high adherence to PrEP is critical to its efficacy (7–11). This result also supports our continued efforts to pursue this innovative biomedical approach to address significant risk of acquiring HIV as experienced by many PWUD in treatment. It should be noted that prior research has documented disengagement from PrEP services to be substantial after a brief period of participation in PrEP trials and HIV infection rates during gaps in PrEP use is found to be high (27). These results thus suggest the need to monitor and provide support for long-term adherence given that participants in the current study have been on PrEP for just over a month. Furthermore, these results should be interpreted with caution, given that self-reported adherence to PrEP may not always correlate strongly with actual drug levels (28).

Our qualitative data further suggest that higher PrEP adherence in our sample may have been due to various facilitators of medication adherence. For example, participants reported the use of memory aids (e.g., alarms, text messaging) as a potential tool to improve adherence. This is consistent with prior findings, where the use of mobile technologies (mHealth) has been shown to significantly improve medication adherence in vulnerable population (29–31). In contrary to prior findings (32, 33), all participants in the current study reported no out-of-pocket costs related to PrEP (e.g., medication, clinic visits), thus facilitating their PrEP uptake and adherence. It is encouraging that most private and public insurance plans in the US currently cover the cost of PrEP. Similarly, many participants indicated the perceived benefit of PrEP use as a reason for adhering to PrEP use. Prior studies reported similar findings, where individuals were willing to use PrEP with higher efficacy in preventing HIV (34, 35); no such studies exist for PWUD. Furthermore, support from social networks, for example family and friends, tended to positively impact participants' adherence to PrEP, and this is consistent with prior studies (36, 37). Interestingly, with PrEP use, partner support in discordant relationship was significant. This is important given that sero-discordant couples are considered to be at high-risk of HIV transmission, and could significantly benefit from increased uptake and adherence to PrEP (12).

Not surprisingly, our findings lend support to previous research highlighting the side-effects as one of the potential barriers for PrEP use. Previous studies have shown potential side effects from PrEP medications as being one of the major barriers to uptake (32, 35, 38, 39), yet numerous studies suggest that currently approved PrEP medications have few or no perceived side effects (7–11). Similarly, participants' concerns about social stigma corroborate previous research identifying PrEP-related stigma as limiting the individual and public health benefits of PrEP among at-risk individuals, including PWUD (40, 41). Many participants expressed concern that taking PrEP medication could lead others to believe they are HIV-infected and on an ART regimen, which negatively influenced their adherence to PrEP. Furthermore, participants perceived the need for daily use of PrEP as a potential barrier to adherence. Contrary to national recommendations (12), participants in this study preferred if PrEP could be taken on an as needed basis. This finding aligns well with a recent finding which has documented *on-demand* PrEP to be effective in reducing HIV transmission (42, 43). Additionally, developing methods of delivery other than daily oral dosing, such as long-acting injectable PrEP (44), may minimize adherence concerns in the future.

Researchers, clinicians, and community members have often raised concerns that PrEP could lead to an increase in high-risk behavior because of a reduced perception of HIV risk (25). Interestingly, participants in the current study overwhelmingly reported no increases in risky behaviors while on PrEP. It should be noted, however, that almost two-thirds of participants shared injection equipment and less than half of participants reported consistent condom use and, overall, they tended to maintain this pattern while taking PrEP. Given that a significant proportion of our sample exhibited risky behaviors, continuation of such behaviors may put these individuals at ongoing risk for HIV infection despite being on PrEP. These data suggest that although PrEP use alone is not associated with risk compensation, PrEP users are at high risk for HIV infection and require complementary behavioral HIV risk reduction strategies.

The conclusions drawn from this study are not without limitations. As these results are specific to high-risk PWUD in a substance abuse treatment setting, our findings may not be generalizable to other risk populations or PWUD in other settings. In addition, we relied on self-reported measures to assess participants' experiences, perceptions, and behaviors, which may have resulted in them under- or over-reporting certain behaviors (e.g., drug- and sex-related risk behaviors, PrEP adherence). Furthermore, the sample size limited our ability to observe potential differential patterns among sub-groups, specifically by age, sexual orientation, or race/ethnicity. Regardless of these limitations, we believe that our findings carry important implications for efforts to improve the PrEP continuum of care among high-risk PWUD in the US, where PrEP for primary HIV prevention still remains understudied and underutilized.

## Conclusion

To our knowledge, this is the first study to explore the experiences and attitudes related to PrEP uptake among PWUD in a substance abuse treatment setting. The results from the current study provide preliminary evidence supporting the successful integration of PrEP within a MMP—a common type of drug treatment setting—where high risk PWUD can be readily reached. The findings support our continued efforts to offer PrEP to high-risk PWUD as an important part of a combined HIV prevention package (4–6). Overall, we believe our findings have encouraging implications for future public health research and for health promotion interventions, practices, and policies for PWUD, particularly in real world treatment settings.

## Author contributions

RS and MC conceived the study design. RS led data collection. Both RS and MC conducted statistical analyses and supported interpretation of results. RS wrote the first draft of the manuscript. Both the authors provided considerable editing, revisions, and content review of the initial manuscript draft and approved the final draft of the manuscript.

### Conflict of interest statement

The authors declare that the research was conducted in the absence of any commercial or financial relationships that could be construed as a potential conflict of interest.
